# Supplementary material for: Complexome profiling of the Chlamydomonas psb28 mutant reveals TEF5 as an early PSII assembly factor
Source: Plant Cell. 2025 Mar 17;37(6):koaf055. doi: 10.1093/plcell/koaf055 (PMC12164590; doi:10.1093/plcell/koaf055)
Supplement: koaf055_Supplementary_Data [file koaf055_supplementary_data.zip › psb28_interactive.zip!/plots/intensity/qualityPlots/peptides/PSAG_Cre12.g560950.t1.2.html]

### Cre12.g560950.t1.2

Trivial name: PSAG  
SeqCoverage [median]: 25.40  
Mass: 13.22 kDa  
  
 

| Sequence | MissedCleavage | Mass [Da] | wtIntensity | muIntensity | Score |
| --- | --- | --- | --- | --- | --- |
| EANFDSTVGPK | 0 | 1163.55 | 1262536.39 | 1144531.91 | 169.41 |
| FVFLGYQR | 0 | 1028.54 | 851707.78 | 699437.91 | 196.32 |
| FVFLGYQRR | 1 | 1184.65 | 0.00 | 1531.19 | 42.11 |
| REANFDSTVGPK | 1 | 1319.65 | 111415.87 | 107298.20 | 172.13 |
| TTGATYFDDLQK | 0 | 1358.64 | 1492780.92 | 1973772.33 | 369.08 |
|  |  |  | 3718441.0 | 3926571.5 |  |
